# Healthcare professionals and commercial milk formula recommendations in the urban Mexican context

**DOI:** 10.3389/fpubh.2023.1260222

**Published:** 2023-11-16

**Authors:** Sonia Hernández-Cordero, Mireya Vilar-Compte, Cecília Tomori, Vania Lara-Mejía, Natalia Rovelo-Velázquez, Keily Hayes, Rafael Pérez-Escamilla

**Affiliations:** ^1^Research Center for Equitable Development EQUIDE, Universidad Iberoamericana, Mexico City, Mexico; ^2^Department of Public Health, Montclair State University, Montclair, NJ, United States; ^3^Johns Hopkins School of Nursing and Johns Hopkins Bloomberg School of Public Health, Johns Hopkins University, Baltimore, MD, United States; ^4^Department of Health Policy and Management, Yale School of Public Health, New Haven, CT, United States; ^5^Department of Social and Behavioral Sciences, Yale School of Public Health, New Haven, CT, United States

**Keywords:** breastfeeding, commercial milk formula, commercial determinants of health, healthcare professionals, marketing, infant feeding practices

## Abstract

**Introduction:**

Breastfeeding (BF) is considered an essential component of optimal care for child health and development. In the past two decades, global data have shown improvements in some, but not all, BF indicators. Despite these positive changes sales and per capita intake of commercial milk formula (CMF) have increased globally. The CMF industry invests millions of dollars in marketing, which targets families and healthcare professionals (HCP). In Mexico, more than half of the mothers (53%) who feed their infants with CMF chose their Brand on the recommendation of HCP. Understanding the reasons behind the current recommendations for the use of CMF by HCP is important for the design of BF interventions. The primary objective of this study was to explore Mexican HCP' beliefs, attitudes, perceptions, and practices about early infant feeding practices, and reasons for recommending CMF. The secondary objective was to explore pregnant women and mother's perceptions of the infant feeding recommendations they received from HCP, and of the factors that influenced their infant feeding decisions.

**Methods:**

The study was based on a secondary qualitative data analysis of a WHO/UNICEF multi-country study. We analyzed focus group discussions (FGDs) and in-depth interviews (IDIs) from Mexico. Data were collected through convenience sampling in Mexico City and Guadalajara. HCP (n = 34) analysis was based on IDIs, and pregnant women or mothers of children 0-18 months (*n* = 74) on FGDs and IDIs.

**Results:**

Through a thematic analysis, we identified the socioecology of BF and triangulated HCP and women's accounts. HCP, pregnant women, and mothers recognized that several factors might have influenced their infant feeding decisions including healthcare facilities' policies and maternal work conditions. Although HCP believed that BF is the best way to feed newborns and young children, they routinely recommended CMF. On the other hand, pregnant women and mothers had a strong belief that BF is the best way to feed their babies. However, when women sought support from HCP, the latter often recommended switching to CMF.

**Discussion:**

This study highlights the discordance between HCP perceptions and mothers' experiences of HCP recommendations about infant feeding. Our findings support a national call for policy actions.

## 1 Introduction

Worldwide, breastfeeding (BF) is considered an essential component of optimal care for child health and development (1). To achieve optimal growth, development, and health the World Health Organization (WHO) recommends that infants should be exclusively breastfed for the first 6 months of life and thereafter receive nutritionally adequate and safe complementary foods while continuing BF until at least 2 years of age (2). Globally, suboptimal BF is estimated to be the cause of nearly 600,000 infant deaths annually from pneumonia and diarrhea alone (3). In the past two decades, global data have shown improvements in some, but not all, BF indicators (1). Exclusive breastfeeding rates increased from 35% (2005) to 42% (2018) in low-income and middle-income countries, although with notable disparities across and within regions and countries (4). Despite these positive changes in BF indicators, sales, and per capita intake of commercial milk formula (CMF) have increased globally, especially in upper-middle-income countries (5, 6). CMF are either liquid or powdered, milk-based or similar, marketed to be suitable for use as a partial or total replacement of breastmilk, specifically to feed children aged up to 3 years (including infant formula, follow-up formula -FUF-, and growing-up milk -GUM- or toddler milk) (7). CMF often times are advertised as requiring bottles with teats to be fed. Importantly, the WHO and national medical organizations concur that CMF is not necessary for children beyond 1 year and may be harmful. Between 2005 and 2019, total global CMF sales grew by 121.5% (from 3.5 to 7.4 kg/child), and a further increase of 10.8% is forecast for 2024 (8). Conversely, exclusive breastfeeding only increased by 20% in the same period (9). In Mexico, an inverse relationship has been reported between exclusive breastfeeding trends, in infants under 6 months of age, and increased intake of CMF (10).

There is a worldwide transition in infant and young child feeding toward diets with a higher content of CMF (11). Some countries (e.g., Australia and the United States) experienced a decline in BF and a normalization of CMF feeding during the mid-20^th^ century. Such transition is related to urbanization, higher incomes, lack of maternity and work protections, pervasive marketing of CMF, and the failure of policies to protect, promote, and support BF in these new contexts (12). Market reports describe a worldwide CMF “sales boom,” with the majority of sales corresponding to a small number of companies (13). According to the WHO/UNICEF multi-country study, the CMF industry generates US$ 55 billion annually by investing hundreds of millions of dollars in marketing strategies and tactics that systematically sabotage parents' infant feeding decisions (14). Marketing preys on expectations and anxiety around feeding by positioning CMF as a better alternative to BF. This is done through aggressive and sophisticated marketing techniques targeting families and healthcare professionals (HCP) (1, 6).

Around the world, HCP are the main source of BF education and advice, and HCP perceptions of BF are important determinants of women's BF outcomes (1, 15). HCPs can play a pivotal role in supporting BF, but if they provide inaccurate guidance or have negative perceptions of BF, they can undermine mothers' confidence and cause them to question the value of BF (1, 6, 16, 17). Formula milk companies seek to exploit this relationship of trust by actively targeting HCP as part of their marketing campaigns. In Mexico, more than half of the mothers (53%) who feed their infants with CMF chose their brand based on the recommendation of a HCP (14).

Although Mexican regulations state that CMF manufacturers are not allowed to contact HCP, prior studies reported that 15.5% of HCP were contacted (18). CMF brand representatives approach different types of HCP (e.g., pediatricians, nurses, and dieticians) and hospital administrators in public and private healthcare settings (14). In countries such as Mexico, visits are more frequent in private healthcare facilities, as attempts at contact in public hospitals have decreased in recent years which might be partially explained by local regulations (18). Meetings with representatives are often presented as learning opportunities about products and infant nutrition with a range of incentives (e.g., research funding, coupons, ambassadorial roles, merchandise, gifts, and all-expenses-paid promotional trips) (14). Sometimes, specialized milks (e.g., milks for allergies and intolerance), are used as a gateway to reach and influence HCP without scientific evidence to support claims about the products.

Given that HCP have significant credibility on infant health and feeding recommendations among parents and family members, it is paramount to ensure that HCP have the necessary knowledge to support BF appropriately, including avoiding any conflicts of interest (19). This requires understanding their ethical responsibility for not recommending the use of CMF when the mother has stated that she wants to breastfeed, and when there is no medical reason to do so (20). Understanding the reasons behind the current recommendations for the use of CMF by HCP is important for the design of interventions aimed to improve their ability to promote, protect and support BF. The primary objective of this study was to explore Mexican HCP' (1) beliefs, attitudes, perceptions, and practices about early infant feeding practices, and (2) reasons for recommending CMF to mothers. The secondary objective was to explore pregnant women's and mothers' perceptions of the infant feeding recommendations they received from HCP, and of the factors that influenced their infant feeding decisions.

## 2 Material and methods

### 2.1 Study design

This study was a secondary qualitative data analysis of a cross-sectional study. The data comes from a WHO/UNICEF multi-country study that included eight countries, including Mexico, across four world regions (14). The overall aim of the primary study was to document the CMF industry marketing playbook. The study included focus group discussions (FGDs), and in-depth interviews (IDIs) to identify HCP', pregnant womens', and mothers' opinions and attitudes about CMF products and corresponding marketing practices. This research paper describes the results from Mexico using data from IDIs with HCP from public and private healthcare facilities, as well as from FGDs and IDIs with pregnant women and mothers of children under 18 months of age at the time of the study.

### 2.2 Research settings and participants

Data were collected through convenience sampling in two large metropolitan areas, Mexico City (capital and largest city) and Guadalajara (the second largest city). These cities were selected due to their population size and geographic and socioeconomic diversity. Within each of the cities, study areas (municipalities and neighborhoods) were selected based on their contrasting aggregate socioeconomic status (e.g., low, medium, or high socioeconomic level).

A total of 34 HCP were recruited for the IDIs, 17 from each city. HCP (e.g., doctors, consultants, midwives, nurses, childcare professionals, dieticians, and infant feeding supporters) were included if they worked in public or private healthcare facilities, and if they had regular contact (3+ times a week) with pregnant or new mothers (with infants aged 0–12 months). The recruitment was done through different strategies including visits to previously selected health facilities from a list of public and private local facilities, a database of health professionals, and snowball referral sampling recommending another health professional for interview.

Furthermore, a total of 74 women at least 18 years old, pregnant or mothers of children 0–18 months were recruited for the FGDs and IDIs through a combination of on-street recruitment and snowball sampling methods. Women were selected based on predefined sample quotas considering stage of pregnancy, infant age, marital status, socio-economic status, ethnicity, and infant feeding intention and practice. Women were included if they were pregnant for at least 3 months, at least 18 years of age and intending to feed the baby with breastmilk and/or CMF. Pregnant women were excluded if they had complications during gestation.

### 2.3 Data collection

IDIs and FGDs for HCP, pregnant women, and mothers, were conducted in-person between February 2020 to February 2021. A total of 10 FGDs and 44 IDIs were conducted (Table 1). All IDIs and FGDs were recorded and transcribed verbatim and translated into English for the data analysis. Focus groups and interviews were conducted by staff trained in these types of information-gathering tools. Additionally, during the data collection, field supervision was carried out to ensure adherence to the protocol. Transcriptions were kept on a secure server from M&C Saatchi World Services (commissioned by WHO to develop the study) and at *Universidad Iberoamericana*, in Mexico.

**Table 1:** Qualitative research methods by target groups.

**Method**	**Target group**	**Number of participants**
***Health care professionals***		
In-depth interviews (*n* = 34)	• Doctors/consultants: private and publics hospitals • Nurses: private and public hospitals	34
***Pregnant women and mothers of children 0-18 months***		
Focus group discussions (*n* = 10)	• Pregnant women (3+ months pregnant) • Mothers of infants 0–6 months exclusive/predominantly BF from birth • Mothers of infants 0–6 months who started CMF from birth • Mothers of infants 0–5 months who breastfed and then introduced CMF • Mothers of infants 6–18 months who breastfed and then introduced CMF 6–18 months	62
In-depth interviews (*n* = 10)	• Mothers of infants 0–18 months who breastfed or use CMF	10

#### 2.3.1 Healthcare professionals

##### 2.3.1.1 In-depth interviews

The IDIs consisted of individual interviews lasting about 1-h. These interviews were designed to understand the perceptions of HCP on infant feeding practices, as well as their interactions with, and influences from, CMF companies. Interviews were conducted in-person at a place and time convenient to the participant. The IDI guide can be found in Supplementary Data S1.

#### 2.3.2 Pregnant women and mothers

##### 2.3.2.1 Focus group discussions

The key objectives of the FGDs were: (1) to investigate women's opinions on infant feeding practices and their main sources of influence; (2) to understand women's motivation, and the key factors influencing their infant feeding decisions, and (3) to examine women's perceptions of CMF brands, and the emotional resonance of CMF messaging and products. Each FGD lasted about 90 min with 5–7 participants, resulting in a total of 62 participants across the 10 FGDs (Table 1). The socioeconomic status of the women participating in each FGD was relatively homogeneous, to facilitate the comfort and ease of the respondents. The FGD guide can be found in Supplementary Data S2.

##### 2.3.2.2 In-depth interviews

The target group for IDIs was only mothers, and not pregnant women, as the goal was to ask detailed questions on infant feeding practices, with a special focus on better understanding maternal attitudes, norms, and aspirations on this topic. The interviews took place in the everyday environment of mothers (e.g., mothers' or relative's home) with an average duration of 90 min. The IDI guide can be found in Supplementary Data S3.

### 2.4 Qualitative analysis

De-identified transcripts were coded by a team of three researchers (VL-M, NR-V and KH) using *Dedoose* (9.0.86 version). We identified themes following a thematic analysis (21), by repeatedly reading transcripts and triangulating with previous literature on the determinants of BF. Researchers independently coded the initial transcripts and then met to review codes and reach consensus on the coding structure before coding the remaining transcripts. Disagreements between coding researchers were brought up to two senior reviewers (SH-C and MV-C) who facilitated reaching consensus. We discussed the themes with the entire research team and mapped them onto the levels of the socio-ecological model of BF (22). In the case of HCP, analysis was done by type of HCP and public vs. private healthcare facilities. For pregnant women and mothers, their children's age and infant feeding practices were taken into account for the analysis. No differences were identified in terms of type of health center, age of children and infant feeding practices among the included population, so general results are presented, with some exceptions noted where necessary.

To gain a deeper understanding of HCP recommendations, we mapped the themes to the socio-ecological model. We also triangulated the themes identified in HCP IDIs with those in the FGDs and IDIs with pregnant women and mothers and looked for areas of concurrence as well as discordance between them.

### 2.5 Ethical considerations

Before beginning data collection, the study protocol was granted ethical approval by the World Health Organization Research Ethics Review Committee (WHO ERC) on 6 August 2019, and by the Research Ethics Committee of the National Institute of Public Health (Comité de Ética en Investigación del Instituto Nacional de Salud Pública, in Spanish) in November 2019. All participants signed a consent form, which included a description of the study and the implications of participation, using clear and accessible language. To guarantee the confidentiality and anonymity of the information, each interviewee and FGD was identified with an ID that replaced her or his name in the transcript. This research adhered to the ethical principles for research of the Belmont Report, which are: respect for persons, beneficence, and justice (23).

## 3 Results

The study population included HCP (*n* = 34), pregnant women (*n* = 12) and mothers of children under 18 months (*n* = 60). The interviewed HCP were from different specialties, including nurses (*n* = 14), obstetrician-gynecologist (*n* = 9), pediatricians (*n* = 5), general physician (*n* = 4), and neonatologist (*n* = 2), with a similar distribution between public and private healthcare facilities. Mothers participating in the study were included by type of infant feeding practices, and specifically for FGDs, according to their child's age [under 6 months (*n* = 37) and 6–18 months (*n* = 13)] and socio-economic level (low/medium-low (*n* = 30), and medium/medium-high (*n* = 32) income) (Table 2).

**Table 2:** Number of healthcare professionals by specialty and healthcare facility (*n* = 34), and pregnant women and mothers by type of infant feeding practices and children's age (*n* = 72).

***Healthcare professional (In-depth interviews)***				
	***Healthcare facility***			
	**Public**	**Private**	**Total**	
Neonatologists	-	2	2	
Pediatricians	4	1	5	
Nurses	8	6	14	
Obstetricians/Gynecologists	3	6	9	
General physicians	2	2	4	
Total	17	17	34	
***Pregnant women and mothers (Focus group discussions)***				
	***Infant feeding practices***			
	**Predominant Breastfeeding** ^a^	**Formula feeding** ^b^	**Breastfeeding first and currently formula** ^c^	**Total**
Pregnant women (>3 months of pregnancy)	-	-	-	12
**Mothers of babies:**				
0–6 months	12	13	12	37
6–18 months	-	-	13	13
Total	12	13	25	62
***Mothers (In-depth interviews)***				
	***Infant feeding practices***			
	**Breastfeeding**	**Formula feeding**	**Breastfeeding first and currently formula** ^c^	**Total**
Mothers	8	2	-	10

a Mothers breastfeeding since birth and only occasionally using formula.

b Mothers feeding with formula since birth.

c Mothers breastfeeding at the beginning and introducing the formula later.

### 3.1 Health care professionals

**Figure 1:**
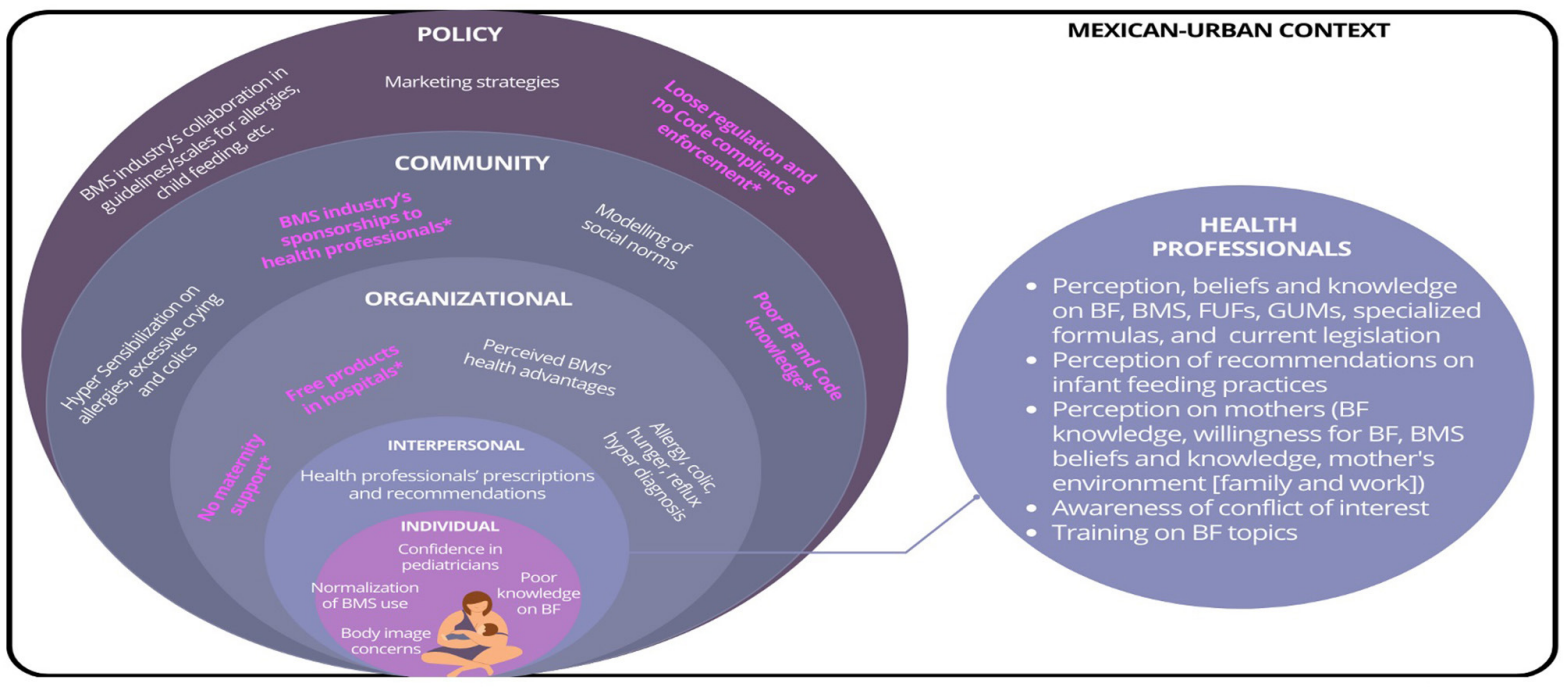
Factors explaining the healthcare professionals practices on infant feeding recommendations. *In pink, factors identified in the study across the levels of the Socio-ecological Model. Based on: Taren D, Lutter CK. The role of breastfeeding protection, promotion and support in a developing world. In Nutrition and Health in a Developing World 2017 (pp. 361–382). Cham: Humana Press.

In this section we present the mapping of the HCP findings from our analysis to the different levels of the socio-ecological model (22) (Figure 1).

#### 3.1.1 HCP' stated beliefs

##### 3.1.1.1 BF as the best infant feeding practice—But often not enough

Among most of the HCP interviewed, no matter their specialty, there was a common understanding that BF is the best way to feed newborns and infants. They reported that they support BF even when mothers run into challenges.

“*... for example, on the first day they'll be like “I'm not getting milk, I'm not getting milk,” and they get frustrated. But that's what we're there for, to support them with breastfeeding, always telling them that it is essential from 0 to 6 months. Breast milk is the best…”* (In-depth interview- Nurse, Public healthcare facility).

HCP reported that the vast majority of women intended to breastfeed, or at least they planned to do so.

“*...I think that nowadays 100% of mothers prefer to breastfeed their babies as the first option and… well, if it were necessary to use a complement…”* (In-depth interview- Nurse, Private healthcare facility).

##### 3.1.1.2 BF is the best, but not enough—HCP recommendations conflict with stated beliefs

Even though HCP stated that BF is best, they nevertheless recommended supplementation with CMF. Most pointed out that breast milk produced during the 1^st^ h after delivery was not enough to meet the needs of their newborn. Others commented that supplementation in the early months was necessary because mothers did not want to exert themselves when they encountered difficulties with BF.

“*...No [Not enough breast milk in the first days]. Most mothers… unless they are mothers who have already had other babies, they typically have a good volume of colostrum, at the start. But most of them cannot meet their baby's requirements, so… that's why we first to stimulate the mother, in order to increase the volume of colostrum and then the transition and mature milk… but during the first few days very few mothers—unless they're multiparous—that's when they can meet their babies' requirements. But most of them need a complement [...] Yeah, the volume during the first 24 h is very low. It's very low…”* (In-Depth-Interview- Nurse, Private healthcare facility).

“*... No [Not enough breast milk in the first days] [...] Not all of them [...] No [Not enough breast milk in the first 24 hours] [...] We need to complement with formula…”* (In-Depth-Interview- Nurse, public health care facility).

HCP saw themselves as important BF promoters; however, their actions and recommendations on infant feeding practices to mothers and their families were not in line with the current BF recommendations.

##### 3.1.1.3 Beliefs, knowledge, and perception of CMF, including specialized formulas

HCP' interviewed for this study did not recognize their own involvement in CMF feeding. Through all their speech, they demonstrated pervasive adoption of marketing language in their own practice. All different categories of HCP agreed that children over 6 months of age needed CMF. They reported that follow-up formulas -FUFs- (intended for children 6–12 months old) and growing-up milks -GUMs- (for children 1–3 years old) are often used by mothers and family and that they are necessary for the transition to cow's milk, as well to meet the nutritional requirements, which are constantly changing at this age.

“*... Well, I think it's okay to use them in different stages, it's adequate and correct. Because they respond to the patients' different needs, which change according to their age [...] Yes, for the transition [...] Well, they can use it for 1*–*2 months, until they can ingest whole milk [...] If they're necessary, I would. But not in general. Only in specific cases when the mother can't breastfeed exclusively…”* (In-Depth-Interview- Pediatrician, Public health care facility).

*[Use of* FUFs and GUMs*] “... I think it's very adequate because a mother's milk changes as well, it evolves. So, there are children who can't be breastfed– we are now having bigger children, and that's also one of the issues, we have babies who are born bigger. And these children need more food, because they're bigger. We need to give these children the milk and nutrition they need…”* (In-Depth-Interview- Obstetrician-gynecologists, Private hospital).

In some cases, they considered that FUFs and GUMs can provide benefits for the child's health, nutrition, and development.

“*... Well, in my opinion, we're lucky that they exist. Of course, these are different times, but 30, 40 years ago, we didn't even have baby bottles. If you had to be given milk, it was from the barn. And if they boiled it for you, you were lucky. If they added peppermint tea to water it down, or anything they added to the milk, you might have a better chance with it. But many people would just pump the cow, warm the milk up somehow, and feed it to them. And nowadays, to have this organized by stage is a blessing. You have less intestinal pathologies such as enterocolitis, intussusceptions, diarrhea [...] When they can pay for it, I would [recommend the formula]. When they have the financial means to pay for it, yes…”* (In-Depth-Interview- Pediatrician, Public health care facility).

In the above case the HCP perceives the CMFs “organized by stage” as a single, necessary set of products, rather than the CMF that may be appropriately used in place of BF when it is not available, vs. unnecessary products after age one.

For specialized CMF, such as those for specific medical conditions (lactose-free or hydrolyzed milks), most of HCP interviewed considered it a great advance to have formulas designed for special needs, such as allergies or some other developmental problems, as well as for the variety already available in the market.

“*... Well, nowadays we have a big range of formulas… in my case, they were a big support for me. There are special formulas that help babies who are intolerant or who have specific allergies. I think that's been one of the great developments, because previously the milk was just similar to mother's milk and that was it. Not a lot of variety. And nowadays they try—well, it's very hard to make it exactly the same, but now they give babies more protection…”* (In-Depth-Interview- Nurse, public health care facility).

#### 3.1.2 Frequent contact with CMF companies with lack of awareness of conflict of interest

Some HCP reported that they have not had contact with representatives of CMF, but mainly because it is not allowed by the rules of their Institutions, especially those working in public healthcare facilities. However, despite these regulations some HCP were approached by CMF companies and received funding to attend scientific conferences. Among the different types of HCP participating in this study, pediatricians were approached the most by CMF companies.

“*.. Yes, they [company representatives] live at the pediatrician's, they're there every day…”* (In-Depth-Interview- Obstetrician/Gynecologist, Private health care facility).

“*... Yes, they give us samples, but only for their specialized formulas. Like I mentioned, they don't leave samples for stages 1 and 2. It's just for their specialized formulas. And sometimes they'll give us pens, or towels, small things [...] Yes, yes. They do invite us [to scientific conferences] [...] Well, yes, for example, there's one they organize themselves, a “summit,” that's what they call it, so it's the company's “summit” and they give us conferences on breast milk, on protein allergies, lactose intolerance... These are scientific topics. They pay for the lodging and travel expenses, and we go to the conference [...] Yes, yes [attendance to the conference]...”* (In-Depth-Interview- Pediatrician, Private health care facility).

HCP participating in the study did not identify receiving funds or gifts from CMF companies as a conflict of interest. Therefore, they did not avoid relationships with the companies in their practice or in their research.

“*... Well, sponsorship is good, having investigation [research] resources is always good, as long as they have ethical guidelines [...] That the research is based on the truth…“* (In-Depth-Interview- Pediatrician, public health care facility).

#### 3.1.3 Lack of knowledge on CMF marketing legislation

During the interviews HCP occasionally mentioned current legislation related to CMF promotion. However, when asked directly about it, very few were able to explain it. At best, they sometimes knew that they should not receive or provide to women and their families CMF samples, and that they should promote BF, but only in general terms. HCP were consistent in reporting that they lacked training regarding the current legislation on the promotion of CMF.

*[Mexican legislation knowledge] “... Yes [...] Oh no… I don't remember specifically, maybe I'm confused. But no… I don't really know about that. I only know about what pertains to my work center where we can in no way promote formulas nor give samples out to patients. And we need to significantly promote breastfeeding”* (In-Depth-Interview- Nurse- private health care facility).

*[Current legislation training] “...Hmm… about that? No, not really…”* (In-Depth-Interview- Nurse, public health care facility).

#### 3.1.4 HCP perceptions of drivers of infant feeding recommendations

##### 3.1.4.1 Providers blame mothers for use of CMF

Most of the interviewed HCP perceived mothers as having everything they need to breastfeed their babies. However, some blamed the mothers for their unwillingness to breastfeed. They considered mothers impatient or ignorant, perceived that mothers were not convinced about the superiority of BF, or that they lacked a plan for how to feed their babies. HCP also stated that a main reason that women choose to use CMF is maternal body image concerns or vanity.

“*... Like I said, a lot of the time mothers want to give them formula out of vanity… oftentimes out of ignorance… or because simply they don't want to breastfeed. And even if you tell mothers there's nothing like breastfeeding, they should at least try to find a good formula...”* (In depth-interview- Nurse, Public healthcare facility).

“... *Who didn't want to because they said that their breasts would be destroyed, that they were going to look ugly. I told them, it doesn't matter why they wanted to block lactation from the beginning because they said “one, because of beauty, what happens if my breasts become ugly?” and I said “they're not going to become ugly, because pregnancy already changed your anatomy, and they're not going to look ugly but yes, more flaccid…”* (In depth-interview- Obstetrician/Gynecologist, Private health care facility).

Some of these statements about mothers went beyond lack of knowledge about BF to blaming mothers' moral character (e.g., “ignorant,” “vain”).

##### 3.1.4.2 Recognition of structural barriers to BF

###### 3.1.4.2.1 Mothers' work conditions

HCP, as well, mentioned that women's work conditions are an important factor on infant feeding decisions. They perceived that for working women it is easier to formula feed.

“*... Well... from what I've gathered, these are people who, due to their jobs and activities, find it easier to prepare infant formula, and that way they can carry on their other activities and not have to spend that time with the baby [...] It's about their comfort in a way, but it's also about their needs. If they need to work, and if their working hours are 8, 12, or sometimes even more, well, this doesn't allow them to breastfeed…”* (In-Depth-Interview- Pediatrician, Public health care facility).

A number of these statements about mothers' work reflect lack of knowledge or skills to support mothers to continue BF when they return to work after maternity leave.

###### 3.1.4.2.2 Healthcare facilities environment—Lack of breastfeeding-friendly practices at the healthcare facilities

HCP stated that hospital practices made an important contribution to infant feeding decisions. HCP stated that women should supplement their infant-feeding with CMF during the first few days, especially those who had a cesarean section. Hospital staff provide formula as standard of care to newborns, especially those born by C-section, and among those who are perceived as having low milk production.

“*... In the first 2 weeks, in my opinion? It's always mixed feeding [...] This is mostly when they have a cesarean section. When they have a vaginal birth it's not as much of a problem, because they instantly start producing milk, but when they have a C section, it takes a bit longer for them to produce milk, so the hospital starts feeding them formula and then they can't start breastfeeding at home until they're producing milk. So, during the first 2 weeks, I think most of them feed them a mix of formula and breast milk”* (In-Depth-Interview- Obstetrician/gynecologist, private health care facility).

One HCP participant from the study said that when there is a breastfeeding-friendly environment, as in the case of hospitals certified as baby-friendly hospitals, as it is expected, all mothers receive support to initiate BF as soon as possible after birth, and do not distribute CMF as standard of care.

“*... I think it's the whole health system, in our hospital, us pediatricians and the dermatologists and the nurses, the gynecologists [support BF]. Everyone [...] No, it's a policy. This is a “Baby-Friendly Hospital” and as a matter of fact, we can't prescribe formula without first instructing them to breastfeed. We aren't allowed to prescribe formula as our first choice, and we need to justify any situation when we prescribe formula…”* (In-Depth-Interview- Pediatrician, Public health care facility).

### 3.2 Pregnant women and mothers' perspectives of the infant feeding environment and HCP infant feeding recommendations on BF

In this section, we report pregnant women's and mothers' perspectives of the infant feeding information practices that they received from HCP. Similar to HCP, pregnant women and mothers were aware that many factors influence their infant feeding decisions.

#### 3.2.1 The influence of supportive and unsupportive BF environments

Pregnant women and mothers recognized that an important factor for infant feeding decisions is the hospital where they will deliver or have delivered their babies. Thus, even though they had already decided about BF, infant feeding policies in maternity services could facilitate or hinder them from carrying out their BF intentions.

“*... I knew it before I was pregnant, what influenced me was the fact that I heard [about] difficult experiences, that's why I did my research to be well-informed. I didn't want to suffer! Close friends told me they didn't have information. During the second trimester I realized that hospital policies can encourage or discourage that process, I knew that when the baby is born you could help the baby or not. The hospital plays an important role, the policies say that they can feed the baby with formula if the mom doesn't have milk and that makes breastfeeding harder. Breastfeeding was not the problem; the problem was choosing where I wanted to have my baby...”* (In-Depth-Interview- Mother, Predominant BF).

Women also identified that living in an environment where BF is stigmatized in public places influenced their decisions about infant feeding practices.

“*... I wanted to breastfeed him although formula can help me a lot. Sometimes we are not at home and he wants to eat but I don' t bring a blanket with me all the time, that is a disadvantage. If I feed him with formula I can do it everywhere. I think formula is the best option because I can't breastfeed him everywhere. Breastfeeding in public is not well regarded by society. It is better for me to prepare a bottle so I can continue doing my chores…”* (In-Depth-Interview- Mother, Formula feeding).

Workplaces were also identified as key environments that influence infant feeding practices. In most cases mothers had difficulties starting or continuing BF because of lack of support at the workplace.

*[Negative way] “... No, and also, earlier, for instance, I grew up with my mom, at home, and now, it is very hard for mom to be home and to be with the baby all day. So now, you go to work and leave them at daycare, with the grandma or the aunt, so it is very complicated to keep up with what our moms used to do, which was on-demand, and they breastfed us until one and a half years old or two, it is very difficult now. I mean, now, formula, mom goes to work, dad too, and that is it…”* (Focus group- Mother of baby from 0 to 6 months, Predominant BF).

In the few cases mothers reported having received support from co-workers, they considered themselves to be “lucky,” drawing attention to the uncommon nature of such support.

*[Positive way] “... I was obsessed with breastfeeding, like a month before I started to buy different pumps, I tried with one and I saw it was not coming out, then I bought a different one and said “I can take this one with me and it is electrical and I do not need to plug it”, I was thinking in everything, then I bought a lunch box and I was practicing how I was going to do it at my workplace, but I was thinking there were many hours. They [co-workers] said “you have 1 h to breastfeed and you can express your milk here” and I could breast pump every 2 or 3 h, so I kept them in the small cups in my lunch box, I have been lucky, all the people around me have supported me with breastfeeding…”* (Focus group- Mother of baby from 0 to 6 months, Currently formula feeding).

#### 3.2.2 HCP, particularly the pediatrician, considered the “authority” for infant feeding recommendations

For most participating pregnant women and mothers, HCP recommendation was crucial in their infant feeding decisions. If a woman was unsure or required support to initiate or continue BF, recommendations from HCP were key to deciding. Recommending CMF, or, receiving a sample of such products, were critical in making decisions about infant feeding.

“*...At the end of the day my baby is more important, I will do what is best for him. If the pediatrician told me to feed him with formula I will do it then. He is the specialist and he knows best. If the priest tells me to feed him with formula I won't do it! All babies are different!...”* (In-Depth-Interview- Mother, Formula feeding).

“*... For example, he [the pediatrician] did tell me “You know what? Give him this milk, it is really good”, he actually gave me a little can so that… [...] a sample and it was actually very good [...] He only gave me two little cans and one was better than the other…”* (Focus groups- Mother of baby from 0 to 6 months, Formula feeding).

Pediatricians, being considered experts in an infant's growth, development, and nutritional needs, played a crucial role in influencing the decision about whether mothers fed their baby CMF.

“*... I do look for it on my own, but the physician's opinion is the most important. The pediatrician is the person I listen to the most when it comes to my baby's diet, because he looks after my baby's weight, and he knows what she needs. And I'm also doing my part by looking for more information, so I can ask him what he thinks about what I investigated or what I'm interested in [...] He's, well, maybe I don't trust him completely, but he's the one who is more knowledgeable about this…”* (In-Depth-Interview- Mother, Predominant BF).

In most cases mothers reported that HCP recommended CMF. However, when mothers had access to HCP with enough training and skills in BF, they could be of great support to solve the doubts and problems presented by mothers and their families, providing the appropriate information for better decision-making on this issue.

“*... My mom too and the hospital [influence infant feeding], because my baby didn't want to eat at the beginning. He didn't eat the first day. I told them [nurses] to give him formula because he couldn't latch to the nipple, but then the nurses helped me. They explained to me that maybe he couldn't eat, but still, they gave me a breast massage to help me bring the milk out, and then the baby could eat…”* (Focus group- Mother of baby from 0 to 6 months, Predominant BF).

Additional representative quotes from IDIs and FGDs about HCP recommendations to pregnant women and mothers are shown in Table 3.

**Table 3:** Representative quotes from in-depth interviews and focus group discussions (Healthcare professionals, pregnant women, and mothers of children under 18 months).

**Healthcare professionals (HCP)**	
***HCP' perception, beliefs and knowledge about mothers' infant feeding intentions and breastfeeding***	
Infant feeding practices	“*...No all women want to BF: 50% of women plan to BF...”* (In-Depth-Interview- Nurse, Public hospital).
Knowledge about BF	*[Poor knowledge] “...Three months, for most of them. If we're lucky, it's 6 months. But since we see all kinds of people, some of them say they've been doing this for a year and a half, and we tell them to stop. That's something else altogether [...] By then, their milk has less nutrients than “agua de horchata” (rice milk) [...] It depends on what the mother eats and the time she has for it. If you have all the time in the world to breastfeed, awesome, but if not... And sometimes their babies already have grown teeth…Stop!...”* (In-Depth-Interview- Obstetrician/gynecologist, Private healthcare facility).
***Perception of mother' decisions about infant feeding practices***	
Perception on mothers	*[Additional support] “...No, [mothers don't need additional BF support] because once the mothers are discharged, the pediatricians keep a close eye on them, in every sense of the word. Vaccination, growth, development, feeding… everything. And once more, since mothers are financially able, if they have any questions they call the pediatrician and bring the babies in for an appointment…”* (In-Depth-Interview- Neonatal nurse, Private hospital). *[Influencers] “...Those who influence the most, I think it is everyone, both medical staff and relatives [...] It is positive. It is positive, but what I have seen is that women, for example, especially young women, get impatient and no longer want to continue breastfeeding, oftentimes because they work. Even if you explain them everything they can do to save the milk in the freezer, the easiest thing is to quit…”* (In-Depth-Interview- Physician, Public Hospital). *[Reasons to use formula] “... Convenience [...] Well, because they work, because they don't want to... Due to aesthetic reasons, they don't want to look flaccid, because they're lazy and want to sleep more, because they want other people to help feed them, such as their husband, or their mother, their mother-in-law, someone else who's helping out, and they want to sleep, they want to go out and they can't breastfeed comfortably in the street, so all of this can be summarized as convenience…”* (In-Depth-Interview- *Obstetrician-Gynecologist, Private hospital*). *[Work as an influencing factor] “… And currently, the main reason why they don't breastfeed is because the woman has to work. So they're away from home for 6, 8 hours, and that's why they can't breastfeed exclusively…”* (In-Depth-Interview- Pediatrician, Public hospital).
Beliefs, knowledge, and perceptions about CMF, including specialized formulas	*[Use of formulas from stages 2 and 3] “... Most of them do need it. And most mothers do use them…”* (In-Depth-Interview- Neonatal nurse, Private hospital). *[FUFs and GUMs benefits] “... About starter formulas, well, I think breastfeeding is ideal at first. After they turn 6 months old, they're starting to eat other foods, and the weaning period begins. If they want to stop breastfeeding, I'm obviously not against it and I think they can use formula. They're enriched with Omega-3, DHA, and all of these things that have been proven to be useful in terms of children's neuro-development... And follow-up formulas, from 1 year on, are obviously more enriched with calcium, they even have a better taste, and have everything they need for children to accept them. When they're small, they can't tell you what they like and what they don't, but when they're older, they just leave the baby bottle there if they don't like it [...] Yes, of course [babies need stage 2 and 3 formulas] [...] Yes, I would recommend stages 2 and 3…“* (In-Depth-Interview- Gynecologist, Private hospital). *[CMF benefits] “... Um… well, in terms of milk development… well, for babies with special needs, like anti-reflux milks or comfort milks, but those are for—well, maybe they can't tolerate the milk's protein, in some cases…”* (In-Depth-Interview- Nurse, Private hospital).
	*[CMF benefits] “...Well, that'd be the addition of some components, for example, all of them have iron supplements nowadays, DHA, and there's the ones that are hydrolyzed... There's a formula for all types of intolerance, if I had to recommend a formula, it'd be for those who are intolerant, so, besides convenience, the second reason why they stop breastfeeding is because the babies are intolerant [...] As a result of our current needs. In the past, and I'm talking about when I was young, 40 years ago, we obviously didn't have such advanced formulas. There was the classic one, NAN, and maybe one and two more, and that was it. But nowadays, no matter where you look, you can see more than 20, there's an extensive variety, there's some with more iron, with less iron, Comfort, the ones with Soy, hydrolyzed ones, there's a vast number of them, and they're all in the market because they have certain benefits for the babies. And nowadays, babies are intolerant and have allergies to everything, so the market had to develop formulas that respond to these needs. That's the advantage formulas have nowadays…”* (In-Depth-Interview- Gynecologist, Private hospital).
Contact of CMF companies and awareness of conflict of interest	*[CMF companies contact] **“**… And actually a while back I worked at private hospitals and they still do that, “here's your ration of tubs, here's your liquid milk”… it's almost always liquid, then some in powder, and all the usual gifts… baby bags, mugs, photo albums and things like that. Luckily it's not like that in the public sector, so it's good for me, for the babies, and for the moms..”* (In-Depth-Interview- Nurse, public hospital). *[CMFs companies sponsorship] “… Well, everything's valid, depending on the intention. And their intention is always to sell. So, if they offer you money and ask you to focus on certain aspects and help them promote their products... Well, they're setting your path for you, and you have to decide if you want to follow it or not. But as a pediatrician, you know what you can recommend and what you can't. For example, talking about Nestlé... Who's going to prescribe Gerber? I mean. But their formulas are useful, of course they are [LAUGHS]. And you'll be helping nourish that baby, you'll help them gain weight, have less reflux, not have spots on their skin due to dermatitis…”* (In-Depth-Interview- Pediatrician, Public hospital). *[CMF companies sponsorship] “… Well, I think it's okay as long as it's a 100% scientific event, so that science can be advanced, I think that's correct...”* (In-Depth-Interview- Pediatrician, Private hospital). *[CMFs companies sponsorship] “... Everything meant for research and nutrition is excellent…”* (In-Depth-Interview- Physician, Private hospital).
***Healthcare facilities environment***	
Breastfeeding-friendly practices at the health facilities	“*... To mothers who– If they have a specific prescription due to which they need to formula feed their baby, we have the ability to write them prescriptions so they can get their formula. But it has to be justified. For us to be able to give them formula, they need to have a concrete prescription, and the mother needs to have tried and prove she's unable to breastfeed– she either needs to have galactosemia or the baby should has an illness and can't drink lactose, and in that case, we have drugs here in the pharmacy– I mean, we have special formulas in the pharmacy, and we give that to them…”* (In-Depth-Interview- *Pediatrician, Public Hospital*).
**Pregnant women and mothers (FGs and IDIs)**	
**Pregnant women and mothers' perspective**	
Infant feeding environments	*[Hospital practices- supporting breastfeeding] “... Yes, in my case, when I gave birth, in the hospital, they did not want to feed him, they always said: “Bring the baby to the mother, it does not matter milk is not coming out” that's how I was able to produce milk, at the beginning, I suffered a lot because I stayed at the hospital for 3 days and my baby cried for a whole day because I did not have milk, but even then they said: “It has to come out, drink water” so it was hard. I said, don't you see what is happening? But you end up thanking them, because they push you to breastfeed, so you should not put yourself any barriers of saying: “I can't”, you can, in my case…”* (Focus group- Mother of baby from 0 to 6 months, currently formula feeding). *[Hospital practices- supporting breastfeeding] “... Because I was working, I was pregnant with twins, only one was able to be born, that is why I had a lot of talks at IMSS (Mexican Social Security), they talked a lot about breastfeeding, all government hospitals are pro-breastfeeding, so, they explained all the benefits of breastfeeding, for the child and for myself, it was a lot, same thing with the nurses there, the doctors, then, my husband went with me to the talks, he was also pro-breastfeeding, and after my baby was born, I looked for a pro-breastfeeding pediatrician and my mom use to tell me “just a little formula” and I told her no, only breastfeeding, so, I actually did not paid attention to my mom and only listened to what I heard at the talks from the WHO, and similar things, it was more input from the hospital…”* (Focus group- Mother of baby from 0 to 6 months, Currently formula feeding).
	*[Workplace environment] “... Yeah, it was my mom at the beginning. I wasn't producing much milk, so my mom would say: “we will have to use formula,” but my sister said, “drink more water, and that will help you to produce more milk. Massage your breast, and insist on it. Don't give him formula.” That's what I did with my first child; I only breastfed him. With my younger child -the girl- I had to use formula because I had to work. I also decided to extract milk and store it, but sometimes I didn't have the time, or I couldn't obtain enough…”* (Focus group- Mother of baby from 0 to 6 months, Predominant BF). *[Workplace environment] “I know many women who stop breastfeeding their babies and substitute it with formula because they work, and they have to leave them in the nursery because there is nobody there to help them, so they stop breastfeeding.”* (Focus group- Mother of baby from 0 to 6 months, Predominant BF). *[Workplace environment] “I breastfed my first child for a year, along with other foods, of course. But with X, I decided I might just breastfeed her until she is 6 months old, because I had to work, but now I'm having second thoughts because she is so little.”* (Focus group- Mother of baby from 0 to 6 months, occasionally use formula).
HCP, particularly the pediatrician, referred as “authority “ for infant feeding recommendations	“*... I feel safe! They know what they're talking about; they know how to explain the information. Other people explain those topics with scientific terms. Pediatricians explain things in a different way; it is easier to get what they say…“* (In-Depth-Interview- Mother, Formula feeding). “*... Only my first baby. We visited the pediatrician because she was constipated and she gave me a white one, it has a bear…”* (In-Depth-Interview- Mother, Predominant BF). “*... In my case, it is my first daughter, whatever the pediatrician says, I have done everything; I mean, the milk he told me was the first one I gave here…”* (Focus group- Mother of baby from 0–6 months, currently formula feeding). “*... Online and I also call my pediatrician [...] To be honest I prefer to call the doctor. I try to verify what he tells me…”* (In-Depth-Interview- Mother, Predominant BF).

### 3.3 Triangulating HCP' and pregnant women's and mothers' perspectives

Both HCP and pregnant women and mothers of children under 18 months recognized that there were several factors that might have influenced infant feeding decisions. Some factors were related to the environment, such as healthcare facilities' policies and maternal work conditions, in the unique complex context of Mexican metropolitan areas (Figure 1).

Focusing on the interpersonal and individual levels of the socio-ecological model (Figure 1), we showed that HCP believed in principle that BF is the best way to feed newborns and young children and see themselves as BF promoters. However, HCP perceived that mothers' decisions about infant feeding were driven by their willingness to breastfeed, the information made available to them, and their personal knowledge and experiences with BF. Some HCP described mothers as impatient, and as having little interest and persistence in BF, so they felt compelled to recommend CMF for the benefit of the baby's health. From the mothers' perspective, HCP recommended CMF because they believed that BF was not enough to fulfill a child's nutrition requirements during the newborn period or later on, they believed that CMF would alleviate allergies, or they felt that CMF would address common baby behaviors that either they or the mothers found problematic.

“*... It was cute at the beginning [Breastfeeding] but I was desperate later on because he was crying a lot, so I went to the doctor. [...] The doctor told me he was crying because he was hungry. They told me to start using formula because otherwise he will continue crying…”* (In-Depth-Interview- Mother, Formula feeding).

In this common scenario, crying is interpreted by the HCP as a sign of hunger and the automatic solution is CMF. No BF support is offered and there is no discussion of the need to breastfeed more frequently, crying as a signal for multiple potential needs (not only hunger) nor how to support a crying baby (other than offering CMF).

“*... I think disinformation plays a role. My mother in law told me that she didn't breastfeed her kids because her doctor said her milk was watery and would not be enough to fill up her baby…”* (Focus group- Mother of baby from 0 to 6 months, Predominant Breastfeeding).

Here the HCP made the incorrect assumption that breastmilk was inherently inadequate and insufficient (“watery”).

“*... It depends on the moment, my daughter was born in IMSS [public hospital], because of the school, and first of all my nipple was not properly formed, so she could not suck well, then, I did not produce much, and they did not insist as much, they [at the hospital] said: “I help you [with breastfeeding]” but they saw it was not working, and she was not big, she was a tiny thing, they said: “I will come back for her” so they used to take her and feed her with formula and brought her back sleeping, and when she woke up, they helped me again, but because they saw again it was not working, they would take her again, they felt very comfortable, they did not insist as much [on breastfeeding]…”* (Focus group- Mother of baby from 0 to 6 months, Currently formula feeding).

In this case HCP lacked the skills to support the mother with BF and made the decision to feed the baby CMF instead.

“*... I trust the pediatrician because my milk wasn't enough to fill him up at night, and this last Saturday I went to the appointment, he told me to start using formula because I told him it had been three straight nights the baby couldn't sleep until 4 or 5 in the morning. My baby was not sleeping, so I gave him two ounces of formula, and he finally relaxed…”* (Focus group- Mother of baby from 0 to 6 months, Predominant BF).

Here, the mother perceived BF as the cause of wakefulness and the HCP “solves” this concern with CMF without addressing any underlying BF issues or explaining that frequent waking and feeding is normal in the first few months.

Pregnant women, mothers, and families, have a strong belief that BF is the best way to feed their babies. This belief comes from recommendations they receive during prenatal visits, mainly in public healthcare facilities, and from popular knowledge about the benefits of BF. Women are aware of the various difficulties they may face to initiate or continue BF. They report that they often receive little support from HCP. Often, when they seek support from HCP, whom they consider to be the most reliable experts on the subject of infant feeding, they are advised to switch to CMF.

## 4 Discussion

To our knowledge, this is the first study in Mexico describing HCP' beliefs, knowledge, and perceptions to better understand their infant feeding recommendations. We used the socio-ecological model (22) as a framework to understand how HCP recommendations are shaped in the context of a market-driven environment that operates within a weak national system of promoting, protecting, and supporting BF. As a unique approach, we triangulated HCP' perceptions and beliefs with those from pregnant women and mothers of children under 18 months, to better understand HCP infant feeding recommendations in practice.

In principle, all HCP agreed that BF is the ideal way to feed newborns and young children. Their beliefs and their lack of training—both about the importance and physiology of BF and in skills to solve common problems on BF—resulted in infant feeding recommendations that do not follow either national or international guidance. HCP justified their recommendation of CMF based on the mistaken belief that BF is nutritionally inadequate or blamed mothers' unwillingness to breastfeed. An important finding of our study is HCP' negative perceptions about mothers. Across the various categories of HCP, there was a perception that women are capable of BF their children, but because of their “ignorance,” “vanity,” “laziness,” or “lack of interest in BF,” they prefer or need to use CMF. These results are consistent with findings of Bueno-Gutiérrez et al., in a study carried out among Indigenous communities in central Mexico (24). The negative perception of mothers by HCP and their beliefs about the inadequacy of BF to meet infant needs is a product of colonial and biomedical frameworks that have been well-documented globally (25). These frameworks, propelled by the authority of colonial governments, played a key role in undermining Indigenous practices of BF and facilitating the widespread use of CMFs often in the name of “improving health.” Our study also confirmed prior reports about poor training on BF topics among HCP, such as in a study conducted among HCP serving beneficiaries of the *Prospera* Program, the largest conditional transfer program in Mexico (26). One of the objectives of that study was to describe BF knowledge and skills among primary physicians, registered nurses and nurse technicians working in clinics that served *Prospera's* population. Vilar-Compte et al. (26), identified a lack of basic knowledge and skills among HCP, to provide adequate BF counseling to mothers living in poverty.

Similarly, in our study, HCP perceived body image concerns as another reason why mothers refused to breastfeed. Specifically, the concern of mothers stems from the appearance of their breasts after the end of the BF period. Increasing exposure to Western cultures, for example, due to the media and immigration, has resulted in the adoption of different behavioral aspects of these cultures (27). Mexico has experienced a rapid nutritional transition in the last decade, which has influenced infant feeding practices by increasing CMF intake (28). Another implication of the Western cultural transition is the exposure to different idealized images of women's bodies. Although body image dissatisfaction is more associated with Western cultures, in recent decades it has expanded to non-Western countries as well (29). A previous study in Mexico showed that greater body dissatisfaction in women is inversely related to the initiation of BF (28). Likewise, one study found that greater body image concerns during pregnancy and after delivery were associated with both the intention to use CMF and its actual use, as well as with a shorter duration of BF (30).

Marketing strategies have recently been described in the Breastfeeding Lancet Series 2023 (1, 6). It is a “multifaceted, sophisticated, well resourced, and powerful system” that has managed to create the need for these products at a global level. HCP play a very important role in the CMF marketing playbook. Through financial support (e.g., commission from sales, invitations to conference events and seminars), corporate-backed science (funding for research) and other strategies (e.g., medicalization of feeding practices for infant and young children), along with the poor training on BF topics, HCP become the main promoters of CMF (6, 31).

Another important result to highlight from our study is the lack of awareness shown by HCP about the implications of receiving funds or gifts from CMF companies and this as a conflict of interest. The HCP participating in this study did not consider these practices as a risk, given their preference for certain brands and their inclination to recommend them to mothers. The most recent evidence shows how conflicts of interest affect the impartiality of HCP and threaten the integrity of their practice (6). Finally, HCP clear lack of knowledge about current legislation in the country facilitates the continued close contact of CMF companies with HCP; consistent with findings previously reported in studies in Mexico and worldwide (32).

Our results emphasize the widespread misperception among HCP that different stages of CMF and GUMs are necessary products. This is relevant in the context of milk formula sales rising globally by 121.5% between 2005 and 2019 (8), which has been largely attributed to GUMs' sales, which grew by 220% over the same period (33). In 1986, the World Health Assembly (WHA resolution 39.28) agreed that GUMs intended for older infants and young children were not necessary for the nutrition and health of young children. In 2019, a group of experts, including the American Academy of Pediatrics and the Academy of Nutrition and Dietetics, also stated that GUMs are not nutritionally necessary and are not recommended for children. Likewise, they expressed concern about the caloric sweeteners used in these products (34). Recent evidence showed that added sugar was found in the ingredient lists of 96 out of 99 (97.0%) GUM products, and most products were further sweetened with non-nutritive sweeteners (33), with this sweetness undoubtedly aimed to appeal to young children's taste preferences (35). In a study analyzing the labels and nutritional content of formulas from 11 different countries, it was found that infant formulas, and particularly GUMs, had a higher content of simple sugars compared to breastmilk, and has been compared to soft drinks in sugar content, as shown in a study in Australia (36), which contains approximately 8 g of sugar per 100 ml (37). Although GUMs products are often fortified with micronutrients that are frequently deficient in the diets of young children in low-income countries, the overall nutritional profile of the products may not be considered suitable for young children, especially given the growing concern about childhood obesity in these same context (38). There is already a well-documented relationship between sugar consumption and risk of obesity and chronic diseases (39). Additionally, there is increasing evidence demonstrating that consuming simple sugars in the early stages of life can result in a greater preference for sweet flavors (40).

Healthcare professionals play a critical role in promotion, protection, and support of BF (1). Consistent with previous studies, infant feeding recommendations from HCP, particularly from pediatricians, had a great influence on mothers and their families in this study (41). According to the womens' experiences in this study, the recommendations of HCP are decisive on how mothers will feed their children. These recommendations can make a pivotal difference in whether mothers decide to initiate or continue BF, or whether they switch to CMF. This has been reported in other studies in Mexico and other countries (15, 20, 42).

Both HCP' and mothers participating in this study acknowledged the presence of multiple factors that influence decisions regarding infant feeding practices. Both groups highlighted issues such as women's work conditions and healthcare facilities' policies as significant driving forces in shaping these decisions. Furthermore, our findings suggest that it is important to consider the context in which the HCP' infant feeding recommendations occur. In Mexico, these recommendations occur within a weak system, with legal loopholes, which fails to protect, promote, and support BF. Noteworthy in this instance is the lack of maternity protection, and a maternity healthcare system that does not adequately invest in training HCP in foundational knowledge about BF or the skills to support it. Hence it is not surprising that women in our study reported that they “lacked support for BF” and “had many doubts about their capacity to breastfeed.” Indeed, in this unsupportive system they often found BF a stressful and exhausting experience. As it has been well documented, CMF companies have developed a deep understanding of these needs and meet them with a combination of “caring” relationships, non-judgmental support, individualized communications, a range of reliable and readily available products, and evocative and reassuring branding (1, 6).

This innovative study allowed us to triangulate and integrate the infant feeding perspectives from HCP, pregnant women, and mothers (from different socioeconomic strata) into the socio-ecological model. Furthermore, the study methodology used diverse methodological approaches including in-depth interviews and focus group discussions, which allowed to collect information at the individual and group levels.

This study also has some limitations which need to be considered. The study took place in two metropolitan areas in Mexico; hence the rural context was not addressed. However, it is important to acknowledge that similar findings have been observed in smaller urban areas as well as rural areas in Mexico and other Latin American countries (24, 42–46).

## 5 Conclusion

Our study demonstrates that despite HCP' believing they are promoting BF, their actual recommendations tend to endorse the use of CMF. The reasons for this discordance are related to their perceptions of women's attitudes toward BF along with their perception of diverse benefits and advantages of CMF, in the context of poor training in BF. Pregnant women and mothers confirmed that HCP had a strong influence on their infant feeding decisions based on advice that was biased toward the use of CMF driven, at least in part, by conflict-of-interest relationships between HCP and CMF representatives. Strengthening the training of HCP in BF and conflict of interest avoidance is urgently needed, particularly given the key role they play among pregnant women and mothers in their infant feeding decision-making.

It is important to interpret the results of this study within a national context in which the system is still not ready to enable the promotion, protection, and support of BF, which unfortunately is the norm in Latin America and the all other world regions. For example, typically the maternity health care system does not enable the initiation and continuation of BF, HCP are poorly trained in BF, maternity protection is weak, and protection of women and their families from CMF marketing practices is limited. Hence, our findings support a national call for policy actions following the socio-ecological model: (1) Governments must strengthen and enforce national WHO Code legislation and the development and implementation of ongoing monitoring mechanisms at the national level. (2) Governments must strengthen maternity benefits for women working in the formal and informal economy sectors. (3) Governments must invest on training HCP. Training should include, among other topics: (a) the fundamentals of infant feeding in general and BF in particular, (b) communication and lactation problem solving skills, (d) CMF companies' marketing strategies, (e) current WHO Code legislation, and (f) what conflict of interest is and how to avoid it. Training, including preservice and in-service training curricula, symposia, and conferences, should target all HCP with a particular focus on pediatricians with the understanding that in the Mexican and other contexts, HCP are considered by parents to be have the most authoritative knowledge about infant feeding. (4) Governments must invest in providing access to baby friendly facilities and BF counseling to all women starting at the time of pregnancy. These programs should include the social support networks of the mother including partners, other family members and friends. These calls to action, requiring implementation through an equity and social justice lens, can strengthen our systems, address gaps and inequities, and help to ensure that women and children receive the benefit of breastfeeding.

## Data availability statement

The data that support the findings of this study are available from World Health Organization (WHO) but restrictions apply to the availability of these data, which were used under license for the current study, and so are not publicly available. Requests to access the datasets should be directed to the authors.

## Author contributions

SH-C: Conceptualization, Formal analysis, Methodology, Project administration, Supervision, Writing—original draft. MV-C: Conceptualization, Formal analysis, Methodology, Supervision, Writing—review & editing. CT: Conceptualization, Formal analysis, Methodology, Writing—review & editing. VL-M: Formal analysis, Methodology, Writing—original draft. NR-V: Formal analysis, Writing—review & editing. KH: Formal analysis, Writing—review & editing. RP-E: Conceptualization, Formal analysis, Investigation, Methodology, Project administration, Supervision, Writing—review & editing.
