# Supplementary material for: Healthcare professionals and commercial milk formula recommendations in the urban Mexican context
Source: Front Public Health. 2023 Nov 16;11:1260222. doi: 10.3389/fpubh.2023.1260222 (PMC10693414; doi:10.3389/fpubh.2023.1260222)
Supplement: Supplementary file 1 [file Data_Sheet_1.docx]

# *Supplementary Data S1. In-depth interview guide - Healthcare professionals*

**INSTRUCTIONS FOR INTERVIEWER**

Before the interviews begin participants must be provided with a participant information sheet, explaining the background to the study and confidentiality. Participants should have opportunity to ask any further questions about the interview before it begins. Participants should be reminded that there are no right or wrong answers and that the aim of the interview is to explore their honest experiences and views about feeding their baby.

For the purpose of this interview, women who breastfeed are including those who feed breast milk to their babies in a bottle, and formula feeding is about women who feed their babies with any kind of baby formula products.

1. ***General***
2. Have you attended any focus groups or interviews in the past 6 months about mothers and babies?
3. Is your work mainly located in the maternity ward or in an office?
4. How often are you in contact with pregnant women or mothers of children 0-18 years old? 3 or more times a week?

***II. Infant feeding practices***

1. In your opinion, do most pregnant women plan whether they will breastfeed or feed their babies with formula?
2. Do you think most women plan how to feed their babies?
3. How do you advise women to feed their babies?
4. Do you take courses to teach mothers during breastfeeding?
5. In your opinion, how do most mothers feed their babies during the first two weeks?
6. How does baby feeding change over the following weeks and months, based on what you have observed?
7. What are some of the tips or practical guidance you give mothers? How do you advise them for breastfeeding?
8. Do you think feeding practices have changed in the past few years? In terms of feeding babies.
   1. What do you think caused this change? What has influenced it?
9. Who influences women’s baby feeding practices? (Physicians, relatives, friends)
10. Thinking of women who breastfeed, what do you think are the factors that help them breastfeed successfully?
11. Do you think that additional support could help more women achieve this successful breastfeeding?
12. Do you think most women can provide enough nutrition to their babies by breastfeeding during the first few days?
13. What do you advise to women who say they don’t have enough milk to breastfeed their babies?

***III. Commercial Milk Formula***

1. Thinking of women who feed their babies with formula, what are the main reasons for their choice?
2. What benefits do mothers who use formula perceive that they are getting?
3. What do you think have been the greatest baby formula developments in the past few years?
4. What do you think about the use of formulas for different stages of development?
   1. For example, the starting ones from 0 to 6 months, the continuation ones from 6 to 12 months, and then growth from 12 to 24 months.
5. Do you think babies need formulas for stages 2 and 3?
6. What do you think of these specialized formulas like the ones you mentioned? (Lactose-free, for cramps, for sensitive stomachs)
   1. Would you recommend them to mothers?
7. Have you seen an increase in women who report that their babies suffer from allergies, including allergies to cow milk?
8. Have you ever recommended any specific formula brands?
9. What are your thoughts on baby formula advertising?
10. Do you think that most women are exposed to baby formula advertising?
11. Do you know if women receive free samples or promotions for baby formula?
12. Have you ever been contacted by a company to promote a brand?
13. Have you ever been approached with free products or samples?
14. Have you ever been invited to events sponsored by baby formula companies?
15. Have baby formula companies ever gifted you material such as rulers or any materials?
16. Do you think that companies who commercialize baby formula products are increasing, or decreasing their attempts to contact healthcare professionals?
17. What do you think of the baby formula companies that sponsor academic departments and research institutions who do research on child nutrition?

***IV. Mexican Legislation***

1. Are you aware of the Mexican legislation on baby formula commercialization?
2. What does this legislation include? Do you know if companies can sell baby formulas and what are they allowed to do and not?

Ok, I will read the law, NOM-222_SCFI / Sagarpa-2018, on sales on powder or dehydrated milk. The legislation establishes that milk formula ads must specifically communicate that the product is recommended only if the baby is intolerant to the mother's milk—

1. Do you think this legislation is being applied?
2. Have you received any training or information on this legislation?

***V. Close***

This is the end of the interview. Would you like to add anything else?
