# Supplementary material for: Healthcare professionals and commercial milk formula recommendations in the urban Mexican context
Source: Front Public Health. 2023 Nov 16;11:1260222. doi: 10.3389/fpubh.2023.1260222 (PMC10693414; doi:10.3389/fpubh.2023.1260222)
Supplement: Supplementary file 2 [file Data_Sheet_2.docx]

***Supplementary Data S2. Focus group guide - Pregnant women and mothers***

**INSTRUCTIONS BEFORE START WITH THE FOCUS GROUP**

Before the focus group begin participants must be provided with a participant information sheet, explaining the background to the study and confidentiality. Participants should have opportunity to ask any further questions about the interview before it begins. Participants should be reminded that there are no right or wrong answers and that the aim of the focus group is to explore their honest experiences and views about feeding their baby.

1. ***General***
2. About sources of information regarding motherhood that you use now when you feel worried and want to know about more things, infant feeding, and child-rearing, which are the sources or channels that you use to obtain information on breastfeeding or formula feeding?
3. Out of these people that you have mentioned, like your relatives, your friends, the doctor, who has an influence? who has been most influential in what you’re planning to do?
4. Does any of you take part in mothers groups or forums on the internet or on any social media platform to obtain information?
5. Which do you think is the best way to feed a baby, from the moment it is born till the baby is 6 months?
6. What about a baby over 6 months and up to one year, how must such a baby be fed?
7. How do you think most women feed their babies? do they breastfeed them or do they give them formula, or what do they do?
8. If we think about different types of milk, not just breast milk or cow milk, but about goat milk, formula, or any type of milk out there, which are the best milk types to feed a baby from 0 to 6 months?
9. What do you think the baby should drink after one and a half year, breast milk, formula, or other milks?
10. How was the experience of breastfeeding your babies?
11. You have already shared with me your own reasons, but if we think about other women, which do you think are the main reasons why women decide to breastfeed their babies?
12. Which are the main barriers and hurdles that some women face for which they can’t breastfeed? What things can prevent a woman, even if she wants to, from breastfeeding her baby?
13. If we think of women who work and things like that, do they face any hurdles?

***II. Formula feeding***

Now let’s talk about formula feeding.

1. What do you think about formula feeding?
2. Do you think that the mother’s diet somehow affects the baby? Can the diet that she has while breastfeeding affect the baby positively or negatively?
3. Which do you think are the main reasons why women choose to formula feed their child? Why do you think some women give their babies formula milk right from birth?

***III. Social media platforms***

​​Now let’s talk about the social media platforms that you use to get information.

1. Which sources of advice and information on infant feeding do you use on-line?
2. Do you feel that what you’re reading is the information you’d like to receive on social media and so on?
3. What about the baby’s feeding or rearing, what topics have you researched?
4. What would you like to see on social media that would help you?
5. What questions do you have about formulas? Is information unclear or what do you think of the industry that commercializes them?
6. Think some formula marketing companies use social media or have any celebrity, artist, or famous mother in their advertising to promote their products?
7. What piece of advertising do you remember from the brands on tv, on street posters, on social media, or any ad]? What pieces of advertising do you remember from formula milks?

***IV. Advertisements activity***

*Now we will show you, __________ is helping us with some advertising examples, both from magazines or publications, and from social media. I’m going to ask you to focus on what these pieces of advertising are conveying to you, while we’re watching. We will stop the images a little for you to do this. Focus on what you like, what you dislike, or what you think is wrong, so we can discuss later on.*

Video #1 nan 3: you have spent day and night making an endeavour when looking after your baby, and it was all worth it to see your baby happy and healthy.

Video #2 nido kinder: nourishing my daughter is as important as protecting her.

Video #3 nido: the stronger from inside, the better to experience.

Video #4 enfagrow: babies need Docosahexaenoic Acid (DHA) everyday for their mental development.

1. What did you like the most?
2. Which one would you choose?
3. When you see these brands and see that there is a wide range in the market, what’s the impact it has on the mother-to-be?
4. Now that you have seen all of these, what came to your mind about the concept of formulas?
5. If there was some advertising to promote breastfeeding, what should it say? Who would have to promote it?

***V. Close***

Thank you very much for having been here and for sharing your opinions.

1. Would you like to add anything to what we’ve just discussed?
