# Supplementary material for: Healthcare professionals and commercial milk formula recommendations in the urban Mexican context
Source: Front Public Health. 2023 Nov 16;11:1260222. doi: 10.3389/fpubh.2023.1260222 (PMC10693414; doi:10.3389/fpubh.2023.1260222)
Supplement: Supplementary file 3 [file Data_Sheet_3.docx]

***Supplementary Data S3. In-depth interview guide - Mothers***

**INSTRUCTIONS FOR INTERVIEWER**

Before the interviews begin participants must be provided with a participant information sheet, explaining the background to the study and confidentiality. Participants should have opportunity to ask any further questions about the interview before it begins. Participants should be reminded that there are no right or wrong answers and that the aim of the interview is to explore their honest experiences and views about feeding their baby.

The interviews should take place in the woman’s natural environment, preferably her home. You should ensure that the woman is comfortable at all times. Participants should be reminded that they can pause or stop the interview at any time. Given the length of the data collection, it is important to make sure the participant has sufficient breaks, and the opportunity to care for her baby when necessary.

This interview should be conducted in two parts. The first section will explore the diary entries the woman has completed. The aim of this is to examine exposure to Breastmilk Substitute (BMS) advertising, and how this makes the woman feel. The second part will explore the mother’s experiences of feeding her baby.

You must judge when and how to move the interview along in enough time to cover all relevant topics**.** You must familiarise yourself with the discussion guide before starting, so that you are aware of questions or themes arising. Ideally the questions should be asked in the order that they are presented. However, it is possible that latter questions may arise at an earlier stage and it is important to maintain the natural flow of discussion. Many of the questions have suggested probes underneath that should be used to elicit more detail depending on the initial response given.

Interviews must be audio recorded on at least two devices, which the participant must agree to prior to the interview commencing. Participants must be assured that their answers will remain confidential

**CONDUCTING THE INTERVIEW**

You should aim to spend around three hours with the mother in her natural environment. During the interview you may take observational notes, such as body language and reactions, and any discussions with others surrounding infant feeding. You must ensure that you record the name and contact details of the participant, the date of the interview, the length of the interview, and your own details in your notes. This information must be kept strictly confidential.

If participants begin to provide information about topics that are irrelevant to the study, it may be necessary to steer them back to the focus of the research- however, this must be done politely and respectfully**.** You must not hurry the interview. If you are unsure of any of the participants’ answers, you must seek clarification, guesses or incorrect information must not be recorded.

After the interview has finished you must complete additional field notes about the interview including any observational notes. For example, you should make notes about:

- Where the baby is and how the mother is engaging with them

o e.g. holding the baby, playing with them

- How often she feeds her baby (if at all) and how she feeds them

- Who else is in the house with the mother

- What feeding accessories are present e.g. brand of formula, bottle, books on infant feeding

**INTERVIEWER - PLEASE COMPLETE THE FOLLOWING**

Interviewer name:

Field supervisor name:

Location of interview:

Area of interview (town and city):

Date of interview (dd/mm/yyyy):

Time of interview (hh/mm am/pm):

**PART ONE: EXPERIENCES OF INFANT FEEDING**

*Thank you for allowing me to come here today for the interview. How are you? How has your week been? Tell me about what you and your baby have been doing?*

*I’d first like to talk about what you have recorded in your diary last week and explore a more about what you saw and how it made you feel. Shall we go through the diary?*

**[Interviewer to look through the diary with the mother]**

**INTERVIEWER READ THE FOLLOWING:**

Firstly, how did you feel about this exercise, did it surprise you in any way e.g. how much information about infant feeding you are exposed to or how little?

I’d like to explore in more detail some of these photos. Which photos do you think stood out to you as the most interesting or that you would like to say more about?

1. Can you tell me a little more about what this is a photo/description of?

- E.g. is it an advert, an email, a display in a shop?

2. Did you give permission to be sent information? Did you sign up to a mailing list or join a club?

3. Looking at the image, what is being advertised or promoted? What age baby do you think that formula is suitable for?

4. What type of mother do you think the advert is aimed at?

- Do you feel it is aimed at you? Why or why not?

5. Did this make you want to buy the product?

- Why? Or why not?

6. Do you have anything else you would like to say about this advert?

7. How are you currently feeding your baby?

8. **Note to interviewer: ask the respondent the appropriate feeding prompt, e.g. for those who are formula feeding ask when they introduced formula.**

- *How long have you breastfed for?*

- *When did you first feed your baby formula?*

- *When did you stop breastfeeding?*

- *Was this how you planned to feed your baby?*

- *If this wasn’t your first child, are you feeding them similarly to your other children?*

9. When did you decide that you’re going to feed your baby this way?

- *If your decision changed when did it change?*

10. What or who influenced your decision on how you feed your baby?

- *Was this what you planned to do?*

- *Did you feel happy in this decision?*

- *Did you feel confident in this decision?*

11. How do the majority of mothers that you know feed their babies?

12. Did anyone advise you to feed your baby in a certain way?

- *If so who?*

13. Where do you seek information on infant feeding?

- *The internet? Books? Parenting sites? Other sources?*

- *If you seek information online what websites do you go to?*

14. If you have a question or concerns about your baby, who do you turn to for information?

- *Do they usually give you good advice?*

- *Who is the most trustworthy?*

15. And do you turn to anyone for support on feeding your infant?

16. What role do health professionals/ experts play when it came to decide how to feed your baby?

17. Do you go to experts first for information or support on how to feed your baby? Or do you get information first and then ask the expert?

- *If yes who? What did they recommend?*

- *Did they recommend any products to you?*

**Note to interviewer: Ask only to mothers who are breastfeeding only**

18. Do you think that there’s anything or anybody that has helped you to breastfeed your baby?

- *Why did you choose not to formula feed?*

**Note to interviewer: Ask only to mothers who breastfed and are now formula feeding**

19. What influenced you to stop breastfeeding?

- *Can you tell me about the moment you decided to stop? Why was this?*

**Note to interviewer: Ask to all mothers who are formula feeding**

20. What, or who, influenced your decision to introduce or start with using formula milk?

- *Did you feel happy in this decision?*

21. Did you introduce formula to your baby for a specific reason?

- *If yes, what was this reason?*

22. **ASK EVERYONE**

23. What do you think is the best way to feed a baby? What a about an older baby around 18 months?

24. What, if any, in your opinion, are the benefits of breastfeeding?

25. What, if any in your opinion, are the benefits of formula feeding?

26. What do you think is the easiest way to feed a baby?

27. Do you think that all women can breastfeed?

28. What signs do you look for in a baby to know that it is feeding well?

- *weight? Height? hunger? sleep?*

29. Do you think that how you feed a baby affects their behaviour at all? Do you think breastfed and formula fed babies behave differently?

30. Do you think there is pressure on women to feed their baby in a certain way?

- *Where does the pressure come from? Who?*

31. Do you have a spouse or partner? If yes, how do they feel about you feeding your baby this way?

32. Has anyone ever recommended formula feeding to you?

- *Did they recommend a certain brand of formula to you?*

33. Has anyone ever recommended breastfeeding to you?

34. Do you think that a woman can breastfeed in public?

35. Do you think that women can breastfeed and still go to work?

- *If yes why?*

36. Have you heard of Stage 2/3 formula before (Continuacion o seguimiento, etapa 2 y 3)?

- *If yes, where did you learn about?*

- *What age is it aimed at?*

- *Is it different to infant formula?*

- *Have you used it before?*

- *Would you use it?*

37. Have you heard of Stage 4 formula before (Crecimiento, etapa 4)?

- *If yes, where did you learn about?*

- *What age is it aimed at?*

- *Is it different to infant formula?*

- *Have you used it before?*

- *Would you use it?*

38. Until what age do you think a baby/child should drink formula milk for? If at all?

39. Until what age should a woman feed a baby breastmilk? If at all?

40. What, if anything, do you think stops women breastfeeding?

41. What, if anything, do you think would help more women to breastfeed?

42. What do you think would make breastfeeding more appealing for women?

43. What do you think would make breastfeeding more feasible?

44. Do you think women in your community have any support when it comes to feeding their baby? If not what supports do you think are needed?

**INTERVIEWER TO TALK THROUGH A FEW IMAGES AND ASK THE FOLLOWING QUESTIONS**

45. Did any of these adverts appeal to you?

- *If yes, why?*

- *Did you buy the product?*

- *If no, why not?*

46. Out of all the photos you have taken this week, which one stands out to you?

- *Which one was the most appealing?*

- *Which one was the least appealing?*

- *Which one was the most useful?*

47. Apart from your diary exercise, can you remember seeing any other adverts for formula milk?

- *If yes what was this advert for?*

- *Where did you see this advert?*

- *What about Facebook?*

48. How often do you see adverts for formula? Regularly? Not very often?

49. Do you like these adverts?

- *What do you think about the images used?*

50. How do adverts make you feel in relation to your experience of being a mother?

51. Do you think that advertising has influenced your decision on infant feeding? Or which products to use?

- *If yes, in what ways?*

- *If no, why not?*

52. Which brand of formula do you use or would you use? How and why did you choose this brand?

- *Are you happy with this brand?*

- *What are the benefits of this particular brand?*

- *What type of mother uses this brand?*

- *Did anyone recommend this brand to you?*

- *Did you discuss or recommend this brand to anyone?*

**IF FORMULA FEED OR BREASTFEED AND FORMULA FEED ASK Q53-57**

53. Would you switch formula brands or products? If so why?

54. Are there differences between formula brands? *Are some brands better than others?*

- *If yes, why are they better?*

- *What makes a brand better?*

- *Are more expensive brands better*

55. Do you read the labels on formula products? What do you look for – ingredients, instructions, advice?

56. Where do you buy your formula?

- *Online? In a shop?*

57. Does feeding formula to your baby impact significantly on your family’s finances?

- *If yes, what does this mean for your family?*

58. Do you access any phonelines, baby clubs, aps or social media sites owned by formula milk companies?

59. Have you ever received any free samples of formula milk?

- *If yes where? Or who from?*

- *Did you use this sample?*

60. Have you received money off coupons for formula? Or been offered free delivery of formula from your local shop?

- *If yes have you availed of this offer?*

61. Have you ever been contacted directly by a formula company?

- *If yes how did they contact you? Was it online? In person?*

- *What did they say?*

62. Do you follow any celebrities, or people you admire, who use formula milk?

- *If yes what do you think of this celebrity?*

- *What products do they use? And what brands?*

63. Do you think formula milk companies should be allowed to advertise their products?

- If yes, who to?

- If no, why not?

64. Finally, have you ever seen any adverts for breastfeeding? If so what did this advert say? Did you like it?

**END OF INTERVIEW**

Thank you for your time, your responses are very much appreciated.

*As stated previously your data will be stored securely. Your anonymised data will be shared with the World Health Organization for public health research purposes. Are you happy with this?*
